# Microbial communities associated with sympagic and planktonic habitats during a polar vortex influencing the North American Great Lakes Basin

**DOI:** 10.1128/mra.00932-25

**Published:** 2025-10-29

**Authors:** Katie M. Owens, Katelyn M. Brown, George S. Bullerjahn, Tijana Glavina Del Rio, R. Michael McKay

**Affiliations:** 1 Department of Biological Sciences, Bowling Green State University 110004, Bowling Green, Ohio, USA; 2 Great Lakes Institute for Environmental Research, University of Windsor 177440, Windsor, Ontario, Canada; 3 Department of Energy, Joint Genome Institute, Berkeley, California, USA; Montana State University, Bozeman, Montana, USA

**Keywords:** Great Lakes, winter microbial community

## Abstract

Microbial communities in Lake Erie and nearby inland lakes were characterized through V4-5 rRNA sequencing during winter 2015, where temperature departures of 12°F–15°F in the lower Great Lakes were recorded. Diatoms were in greater relative abundance in Lake Erie, whereas inland lakes and Sandusky Bay were dominated by flagellate phototrophs.

## ANNOUNCEMENT

Despite a prolonged warming trend (1, 2) with declines in ice cover for the Laurentian Great Lakes (3), cold anomalies occasionally dominate winter weather over the Great Lakes region (4, 5). Winter severity shapes communities’ functional trait composition, and taxonomic composition can differ within a lake between years of varying severity (6, 7). Here we announce data sets that describe microbial community composition in Lake Erie and nearby inland lakes during a polar vortex experienced in 2015 (8).

Mid-winter sampling was conducted in Lake Erie and Sandusky Bay in 2015 (Table 1). Lake Erie sites were accessed by helicopter or represented opportunistic samples during routine ice-breaking operations by the U.S. Coast Guard (9). Nearshore Sandusky Bay, Grand Lake St. Marys, and Lake La Su An sampling targeted areas of active ice fishing. Ice was breached using a SPIRE hand auger (U.S. Ice Drilling Program, Madison, WI). At each location, three replicate holes were augered. Ice cores were rinsed with deionized water, placed in new sealable plastic bags (S.C. Johnson & Son, Racine, WI), and transported to the lab on ice. Surface water was collected from holes using a hand-welded stainless-steel sampling bottle. Biomass from surface water and ice melted at 4°C was concentrated on Sterivex filters (0.22 µm; EMD Millipore, Billerica, MA) and frozen in liquid nitrogen. DNA was extracted using the PowerWater Sterivex DNA Isolation Kit (MO BIO Laboratories, Inc., Carlsbad, CA). Both 16S and 18S rRNA gene sequencing were completed at Joint Genome Institute (Berkeley, CA) on an Illumina MiSeq (2 × 300 bp) (10). Primers 515F-Y (5′-GTGYCAGCMGCCGCGGTAA) and 926R (5′-CCGYCAATTYMTTTRAGTTT) were used to amplify the 16S V4–V5 region with the HotMasterMix Amplification Kit (5 PRIME, Montreal, QC) (11). TAReuk454FWD1 (5′-CCAGCASCYGCGGTAATTCC) and TAReukREV3 (5′-ACTTTCGTTCTTGATYRA) were used to amplify the 18S V4 region (12).

**TABLE 1:** Locations and environmental metadata for sequenced samples^*a*^*^,b,c^*

Sample	Coordinates	Date (mo/day) (2015)	Ice/snow thickness (cm)	NH_3_ (mg L^−1^)	NO_3_ (mg L^−1^)	SRP (µg L^−1^)	No. of libraries	No. of 16S reads	No. of 18S reads	16S reads accession no.	18S reads accession no.	Observed species richness (16S, 18S)
Inland Lake												
Grand Lake St. Marys	40.521, −84.574	01/24	127/0	0.33	1.55	49.0	1	144,020	219,142	SRR34768054	SRR34768079	440, 252
Lake La Su An	41.681, −84.695	02/07	267/12	0.08	0.06	3.0	1	158,201	231,486	SRR34768108	SRR34768077	307, 178
Sandusky Bay												
Sandusky Bay 1	41.487, −82.829	01/19	102/0	0.03	0.11	3.0	1	119,728	197,337	SRR34768101	SRR34768069	255, 261
Sandusky Bay 2		01/28	279/4	0.09	0.10	6.5	1	114,395	130,409	SRR34768100	SRR34768068	326; 258
Sandusky Bay 3		02/18	356/0	0.16	0.54	8.0	2	137,649 139,973	220,531 235,795	SRR34768096 SRR34768095	SRR34768066 SRR34768064	387, 456
Sandusky Bay 4		03/02	406/15	0.27	0.41	23.0	3	128,792 162,400 96,368	211,020 212,547 213,305	SRR34768093 SRR34768091 SRR34768088	SRR34768062 SRR34768060 SRR34768057	324, 277 362, 411 293, 289
Sandusky Bay 4 Ice				0.11	0.07	6.0	3	132,517 145,890 134,894	237,733 213,249 213,341	SRR34768092 SRR34768090 SRR34768089	SRR34768061 SRR34768059 SRR34768058	395, 475 457, 467 383, 298
Lake Erie												
C5	41.634, −81.699	03/04	483/8	0.08	1.13	12.0	2	170,344 137,951	238,224 212,170	SRR34768110 SRR34768087	SRR34768086 SRR34768084	660, 538
EC 1326	41.980, −81.916	03/04	432/11	0.03	0.09	2.0	3	119,141 117,317 147,157	240,682 234,952 242,602	SRR34768076 SRR34768056 SRR34768102	SRR34768083 SRR34768081 SRR34768070	340, 342 338, 309 362, 374
MB002	41.910, −82.915	02/08	305/n.d.	0.06	0.28	3.0	1	136,015	232,328	SRR34768106	SRR34768074	410, 522
MB003	41.859, −82.550	02/09	178/n.d.	0.04	0.42	5.0	1	122,597	188,350	SRR34768105	SRR34768073	217, 214
MB004	41.629, −81.977	02/09	305/n.d.	0.02	0.06	4.0	1	145,133	199,006	SRR34768104	SRR34768072	412, 257
MB005	41.980, −81.916	03/18	38/n.d.	0.04	0.13	0	1	126,958	227,634	SRR34768103	SRR34768071	279, 384

^*a*^ Nutrients were measured at the National Center for Water Quality Research at Heidelberg University (Tiffin, OH) using standard methods (13) and are shown as dissolved (<0.22 µm, Sterivex filtrate) concentrations. Ice thickness and snow accumulation are shown as median values of triplicate measures. Observed species diversity is provided for rarefied data (16S: 31,799 reads; 18S: 92,683 reads), and values were aggregated by sample name with the exception of samples with replicates shown individually in Fig. 1.

^*b*^ SRP, soluble reactive phosphorus.

^*c*^ n.d., not determined.

Sequences were analyzed in RStudio (v.2024.04.2+764) (14) using DADA2 1.8 (v.1.32) (15). Default parameters were used unless otherwise noted. Sequences were trimmed to remove primers and bases with an average quality score of <30 [16S: trimLeft=c(19, 23), truncLen=c(175, 175); 18S: trimLeft=c(21, 23), truncLen=c(225, 225)], error corrected (nbases=2e+08, MAX_CONSIST=15), dereplicated, and merged (16S: minOverlap=20, 18S: minOverlap=15). Amplicon sequence variants (ASVs) were generated, and chimeric ASVs were removed. In total, 5,901 prokaryotic and 4,253 eukaryotic ASVs were created. Between 75% and 84% of 16S and between 69% and 82% of 18S nonchimeric reads remained. 16S taxonomy was assigned from SILVA (v.138.2) (16), and 18S taxonomy utilized the Protist Ribosomal Reference Database (v.5.1.0) (17). Chloroplast and mitochondrial reads were removed (2%–69% reads removed) before using phyloseq (v.1.48.0) (18), tidyverse (v.2.0.0) (19), ggh4x (v.0.3.1) (20), and RColorBrewer (v.1.1-3) (21) to create relative abundance plots.

**Fig 1:**
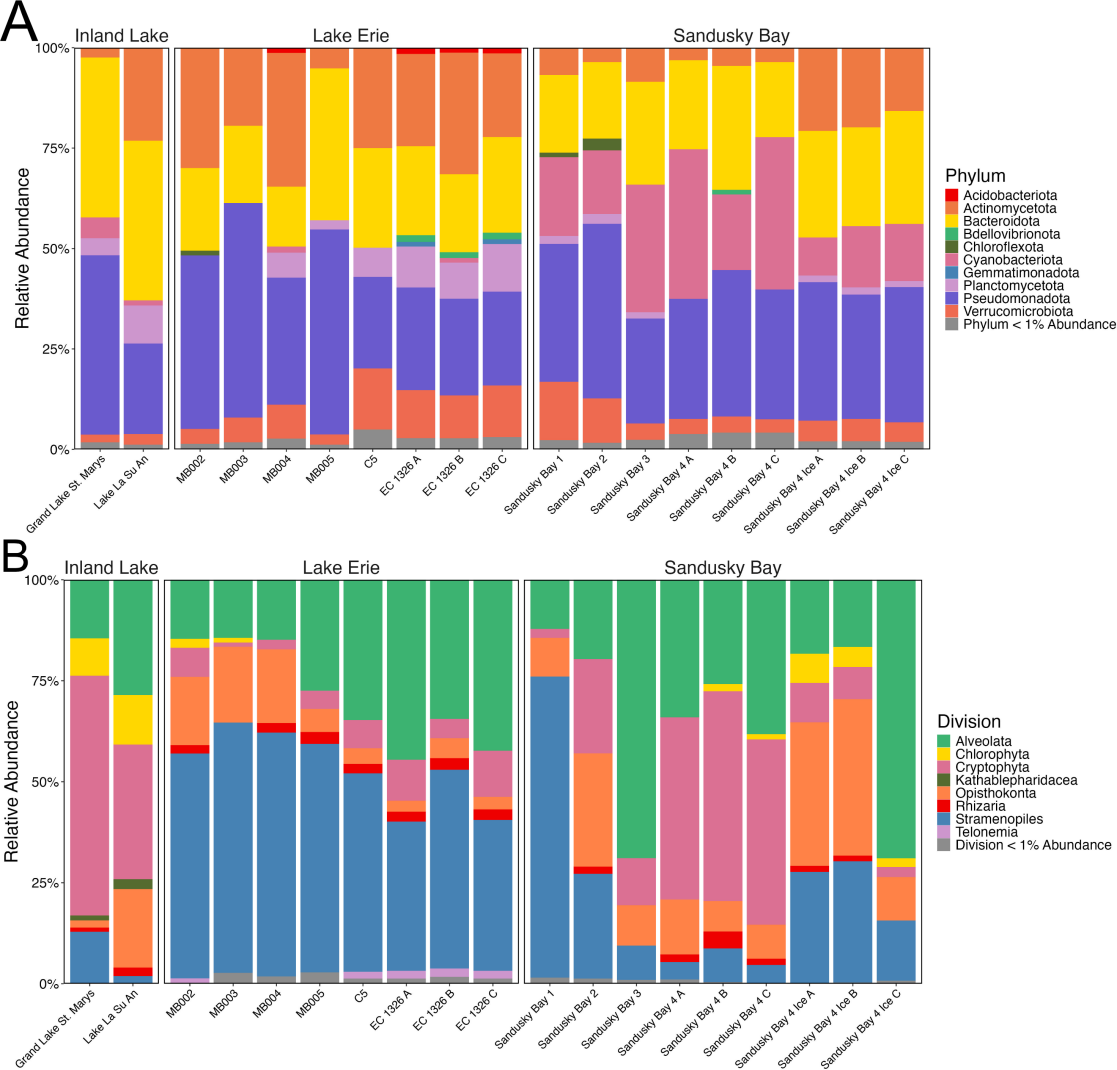
Relative abundance of (**A**) bacterial phyla and (**B**) eukaryotic divisions in Lake Erie, Sandusky Bay, and inland lake (Grand Lake St. Marys and Lake La Su An) samples. Taxa less than 1% abundant were grouped together.

The inland lakes and Lake Erie had similar prokaryotic phylum-level composition, whereas Sandusky Bay had greater representation by Cyanobacteria (Fig. 1A). Stramenopiles were in greater relative abundance in Lake Erie, accounting for over 50% of the eukaryotic community at offshore locations, consistent with previous studies (22, 23), whereas cryptophytes were at high abundance in inland lakes and Sandusky Bay (Fig. 1B). Species richness for prokaryotes (660) and eukaryotes (538) was highest in site C5 (Lake Erie) (24).

## Data Availability

The raw reads from this amplicon sequencing project have been deposited in NCBI GenBank under the BioProject accession PRJNA1298373.
